# Potential role of report nudging on diagnosis and treatment of ventilator-associated pneumonia: a quantitative survey

**DOI:** 10.1017/ash.2025.43

**Published:** 2025-02-17

**Authors:** Kimberly C. Claeys, Blaine Kenaa, Ravi K. Tripathi, Kristen Rayner, Michelle Newman, Surbhi Leekha

**Affiliations:** 1 Department of Practice Science and Health Outcomes Research, University of Maryland School of Pharmacy, Baltimore, MD, USA; 2 Department of Medicine, Division of Pulmonary and Critical Care, University of Maryland School of Medicine, Baltimore, MD, USA; 3 Department of Medicine, Division of Infectious Diseases, University of Maryland School of Medicine, Baltimore, MD, USA; 4 University of Maryland Medical Center, Baltimore, MD, USA; 5 Department of Epidemiology and Public Health, University of Maryland School of Medicine, MD, USA

## Abstract

Diagnosis of ventilator-associated pneumonia (VAP) is challenging and relies heavily on respiratory culture results. The results of this survey underscore the potential for a diagnostic stewardship nudge limiting culture reports to “potential colonization or contamination” in those without clinical findings of VAP to decrease unnecessary antibiotic prescribing.

## Introduction

Over-diagnosis of ventilator-associated pneumonia (VAP) is frequent in the intensive care unit (ICU), often secondary to overestimation of disease probability, nonspecific clinical symptoms, and high mortality rates.^1,2^ Fear of missing a diagnosis, coupled with high rates of respiratory culturing and difficulty interpreting infection from asymptomatic colonization, drives high rates of VAP diagnosis without a compelling clinical indication. This leads to unnecessary antibiotic therapy, which puts the patient at risk for adverse antibiotic events, *Clostridium difficile* infection, and subsequent isolation of multi-drug resistant organisms.^3^

It has been demonstrated that healthcare providers (HCP) order respiratory cultures when there is presence of fever, leukocytosis, and/or hypotension, even without increasing ventilation requirements.^4,5^ HCPs also recognize that the practice of excessive culturing leads to frequent identification of bacteria that can be challenging to differentiate as colonization versus truly pathogenic, especially in patients with worsening non-respiratory clinical symptoms.^1^ With these considerations in mind, we conducted a survey of HCPs to determine potential antibiotic prescribing behaviors in response to a modified respiratory tact culture result report without the identification of bacteria and additional comment suggesting colonization or contamination.

## Methods

From February 10^th^ to March 24^th^, 2023, an electronic survey was sent to HCP practicing in ICUs at the University of Maryland Medical Center to evaluate potential treatment decisions based on lower respiratory tract culture result reports. The survey was created in REDCap and administered via email, with three reminders.^6^ The survey included information on respondent clinical role, years in practice, and primary practice location. Responses were kept confidential, and consent was obtained electronically prior to the survey. Upon completion of the survey, respondents had the option of enrolling in a gift card drawing. The study was approved by the University of Maryland Baltimore Institutional Review Board as minimal risk and consent was obtained within the survey (HP-00082703).

**Figure 1.**
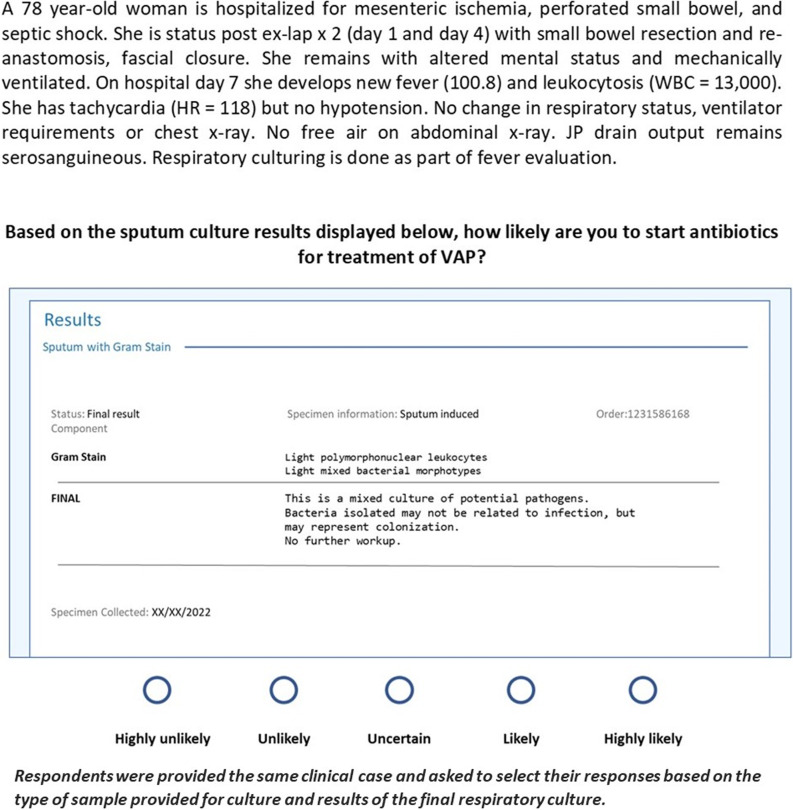
Example survey question stem and mock culture report.

To determine the likelihood of prescribing antibiotics based on culture results, respondents were provided with a single standard clinical scenario with low clinical probability of VAP (Fig. 1), and seven variations of sputum or bronchoalveolar lavage (BAL) culture results in the following order: 1) sputum Gram stain – no organisms, culture with normal flora, 2) sputum Gram stain – few Gram negative rods, culture with normal flora, 3) sputum Gram stain – no organisms, culture no growth, 4) BAL Gram stain abundant Gram negative rods, culture with >10,000 CFU/ml *Enterobacter cloacae* complex, 5) Sputum Gram stain few Gram negative rods, culture with light growth *Enterobacter cloacae* complex, 6) BAL Gram stain Gram negative rods, culture with < 1,000 CFU/ml *Enterobacter cloacae* complex, and 7) the following modified reporting comment without identification of specific bacteria, “Bacteria isolated may not be related to infection but may represent colonization or contamination”. The likelihood of starting antibiotic treatment for VAP was evaluated using a five-point Likert scale from “highly unlikely” to “highly likely.”

We calculated the proportion of HCP who reported they were likely or unlikely to prescribe antibiotics based on the final culture report. We used bivariate logistic regression to calculate odds of antibiotic prescribing for each reporting scenario, relative to modified reporting comment as the reference group. Statistical analysis was completed using SPSS v 29 (IBM Corp., Armonk, N.Y., USA).

## Results

The survey was sent to approximately 250 HCPs, of which 57 (23%) completed the survey. Respondents were residents rotating through the ICU (20, 35%), ICU attendings (16, 28%), nurse practitioners (9, 16%), physician fellows (8, 14%), and clinical pharmacy specialists (4, 7%). One to three years of practice duration was most frequently reported (25, 44%), followed by 3-5 years (21%). The majority reported practicing primarily in the medical ICU (40, 70%), followed by the surgical ICU (8, 14%).

**Figure 2.**
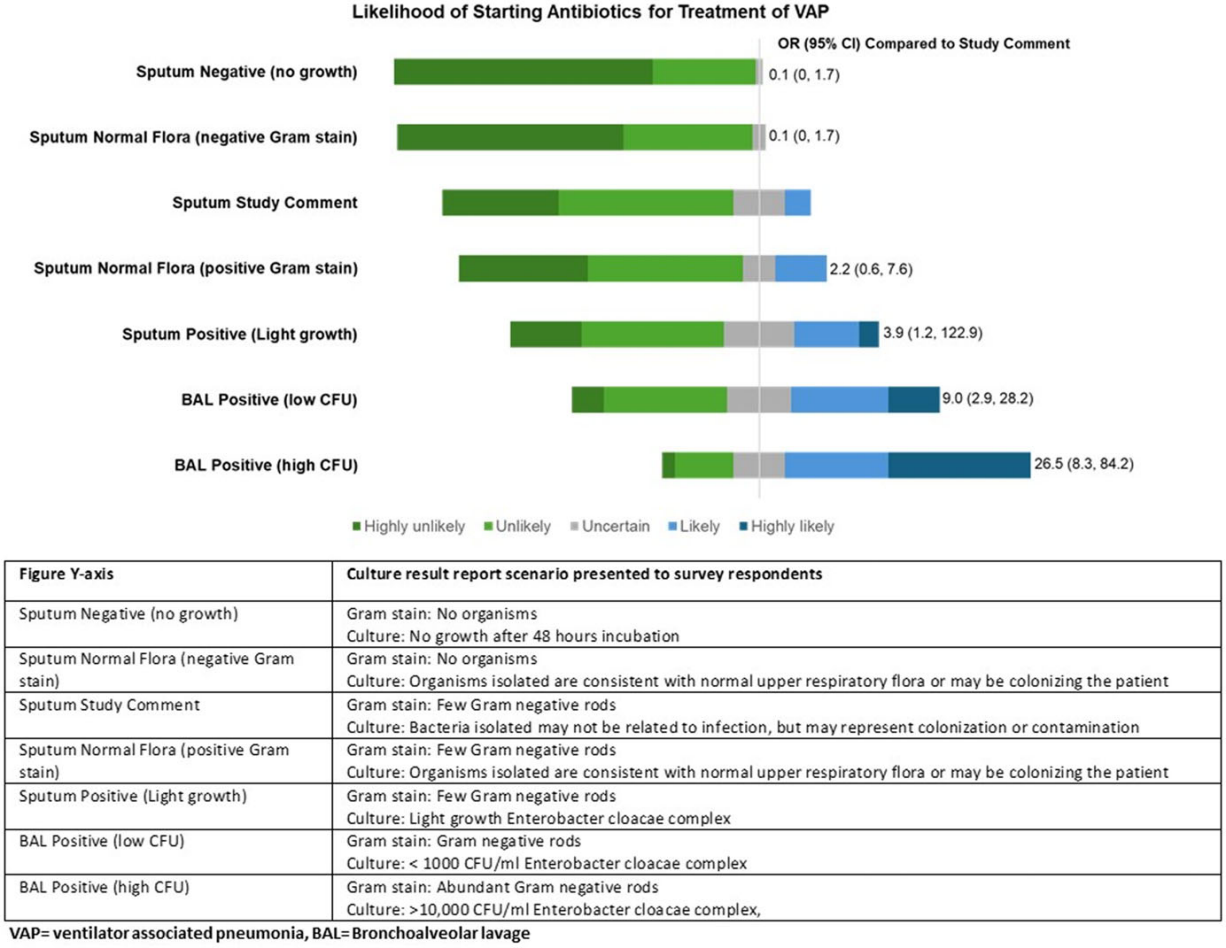
ICU clinician survey responses on likeliness of VAP antibiotic treatment by content cultures reports.

Given the same clinical scenario, respondents were highly likely or likely to prescribe potential antibiotic treatment for VAP when a BAL (67% high cfu, 40% low cfu) or sputum (26%) was reported “positive” with the presence of at least one pathogenic organism, compared to report containing our modified reporting comment (Fig. 2). Respondents were unlikely to initiate antibiotics if a sputum culture demonstrated no growth (61% highly unlikely, 35% unlikely) or the BAL was negative (70% highly unlikely, 28% unlikely). If the respiratory culture reported normal flora, respondents were also highly unlikely (35%) or unlikely (44%) to initiate antibiotics. This was a similar distribution of responses to the modified reporting comment, with 32% highly unlikely and 47% unlikely to initiate antibiotics. The odds of prescribing an antibiotic for VAP in the presence of the modified reporting comment were similar to sputum with no growth and normal flora (OR = 0.1, 0 to 1.7).

## Discussion

In this study using a hypothetical clinical scenario of low clinical probability of VAP, we found that HCP were less likely to treat a patient for VAP when the respiratory culture report included verbiage suggesting potential colonization in lieu of reporting specific organism identification, similar to if the culture report demonstrated only normal respiratory flora. This is consistent with our prior qualitative study where HCP reported a lower propensity toward VAP treatment in the presence of normal flora compared to recovery of a potential pathogen.^1^

Asymptomatic respiratory tract colonization without pneumonia or “asymptomatic bacterisputia” is common, particularly among patients who have been mechanically ventilated.^7^ In the ICU, hypotension, leukocytosis and isolated fever in the absence of respiratory specific symptoms contribute toward respiratory culturing as part of the early sepsis recognition campaign with subsequent identification of the colonizing organisms.^4,5^ The cognitive bias associated with a positive culture – without regard to the underlying clinical scenario – was emphasized in our survey results where we observed a “dose-response” of progressively increasing likelihood of treatment from no growth (least likely to treat) to identification of normal flora, modified reporting comment suggesting colonization, light growth in semi-quantitative culture, low colony count in BAL, and high colony count in BAL (most likely to treat). This is similar to previous survey findings assessing biases in treatment of asymptomatic bacteriuria (ASB) where HCP are more likely to treat a positive culture with certain organisms, even with the same underlying clinical scenario of ASB.^8^ The current survey is limited in generalizability due to use of a single hypothetical clinical scenario and did not include consideration for neutrophil presence or quantification, but findings of antibiotic prescribing bias in VAP are supported by other recent diagnostic stewardship studies.^9,10^ Other limitations include a small sample size and low response rate; however, we believe that this is representative of practice at our, and potentially other academic institutions.

A few studies have reported pragmatically leveraging modification of culture reports to reduce treatment of potential colonization. In a randomized controlled trial, Daley et al., a modified urine culture report resulted in significant improved appropriateness of treatment (80% vs 53%).^11^ This report stated the presence of bacteria but did not identify or quantify the organism, instead instructing clinicians to call the microbiology laboratory if concerns for UTI persisted. In a recent proof of concept study, we found that the modification of the respiratory culture report to suggest colonization results in clinically eligible patients (those with low clinical suspicion of VAP based on a pre-defined algorithm) significantly decreased treatment for VAP (46% vs 18%) without downstream negative sequalae of sepsis or bacteremia.^9^ When combined with clinical judgment and applied to the appropriate patient population, modified reporting of respiratory culture results in patients in the ICU has the potential to be a safe and effective diagnostic stewardship intervention. The results of this survey further support the need to consider how culture reporting can be leveraged to improve diagnosis and management of VAP.

## Data Availability

The data sets used and/or analyzed during the current study are available from the corresponding author on reasonable request.
